# Genetic dissection of heterosis using epistatic association mapping in a partial NCII mating design

**DOI:** 10.1038/srep18376

**Published:** 2015-12-17

**Authors:** Jia Wen, Xinwang Zhao, Guorong Wu, Dan Xiang, Qing Liu, Su-Hong Bu, Can Yi, Qijian Song, Jim M. Dunwell, Jinxing Tu, Tianzhen Zhang, Yuan-Ming Zhang

**Affiliations:** 1 College of Plant Science and Technology, Huazhong Agricultural University, Wuhan 430070, China; 2 State Key Laboratory of Crop Genetics and Germplasm Enhancement, Nanjing Agricultural University, Nanjing 210095, China; 3 Soybean Genomics and Improvement Laboratory, Agricultural Research Service, United States Department of Agriculture, Maryland 20705, USA; 4 School of Agriculture, Policy and Development, University of Reading, Reading RG6 6AS, United Kingdom

## Abstract

Heterosis refers to the phenomenon in which an F_1_ hybrid exhibits enhanced growth or agronomic performance. However, previous theoretical studies on heterosis have been based on bi-parental segregating populations instead of F_1_ hybrids. To understand the genetic basis of heterosis, here we used a subset of F_1_ hybrids, named a partial North Carolina II design, to perform association mapping for dependent variables: original trait value, general combining ability (GCA), specific combining ability (SCA) and mid-parental heterosis (MPH). Our models jointly fitted all the additive, dominance and epistatic effects. The analyses resulted in several important findings: 1) Main components are additive and additive-by-additive effects for GCA and dominance-related effects for SCA and MPH, and additive-by-dominant effect for MPH was partly identified as additive effect; 2) the ranking of factors affecting heterosis was dominance > dominance-by-dominance > over-dominance > complete dominance; and 3) increasing the proportion of F_1_ hybrids in the population could significantly increase the power to detect dominance-related effects, and slightly reduce the power to detect additive and additive-by-additive effects. Analyses of cotton and rapeseed datasets showed that more additive-by-additive QTL were detected from GCA than from trait phenotype, and fewer QTL were from MPH than from other dependent variables.

Heterosis, characterized by Darwin^1^, refers to the existence of superior levels of biomass, stature, growth rate and/or fertility in hybrid offspring compared with the parents^2^,^3^. The rediscovery of heterosis in maize a century ago has revolutionized plant and animal breeding and production^3^,^4^,^5^,^6^. In China, hybrid rice and maize account for approximately 50% and 90% of the total cultivated acreages, respectively. It was estimated that the yield advantage of hybrid maize had contributed an additional 55 million metric tons to the production each year^7^. Although heterosis refers to the F_1_ hybrid, the current knowledge of its genetic foundation is derived from the bi-parental segregating populations but not from F_1_ hybrids. Therefore, it is necessary to dissect the genetic basis of heterosis based on F_1_ hybrids.

Efforts have been made to dissect the genetic foundation of heterosis over the past hundred years^8^,^9^. In early studies, classical quantitative genetic analysis methods were used to analyze the original trait value. As a result, dominance^10^,^11^,^12^, over-dominance^4^,^13^ and epistasis^14^,^15^ hypotheses for heterosis were proposed. In general, dominance includes partial-, complete- and over-dominances, and the epistasis between two loci includes additive-by-additive (*aa*), additive-by-dominant (*ad*), dominant-by-additive (*da*), and dominant-by-dominant (*dd*) effects. The dominance hypothesis for heterosis means that partial-dominance results in heterosis. However, these methods dealt only with the collective effects of all the polygenes. As the introduction of molecular markers and the wide application of quantitative trait locus (QTL) mapping analyses, the dominance^16^,^17^, over-dominance^18^,^19^,^20^,^21^ and epistasis^17^,^22^,^23^,^24^ hypotheses were also supported and these analyses were performed for two kinds of dependent variables, i.e., trait phenotype or mid-parental heterosis (MPH)^22^,^23^,^25^,^26^. In hybrid breeding for heterosis utilization, a genetic mating scheme is usually used to identify elite parents and hybrid combinations through the analyses of general combining ability (GCA), and specific combining ability (SCA), respectively. Recently, an association mapping approach was used for dependent variables such as GCA and SCA in triple testcross and North Carolina III mating designs^27^,^28^,^29^,^30^,^31^,^32^,^33^. The North Carolina II (NCII) mating designs based on different base populations, such as BC_1_F_8_^34^, recombinant inbred lines^35^ and introgression lines^36^,^37^, were reported, and a comparison across different base populations was also conducted^38^. However, the comparison with the differences in the genetic components of the trait phenotype, GCA, SCA and MPH has not been reported, especially for the existence of epistasis.

In this study, trait phenotype, GCA, SCA and MPH in a subset of the 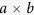 F_1_ hybrids, named a partial NCII mating design, were analyzed by an association mapping approach under an additive-dominant-epistatic genetic model. All the main and epistatic effects for each dependent variable were estimated by the fast empirical Bayesian LASSO (EBLASSO) method^39^. Our purpose was to compare the differences in the genetic components of the above four dependent variables for heterosis. In addition, the effect of the ratio of the number of F_1_ hybrids to the total number of parental lines and F_1_ hybrids in mapping population on association mapping was also investigated.

## Results

### Association mapping for micronaire in cotton and for length of main raceme in rapeseed

#### LD score regression analysis

The estimates for regression intercept were −6.05 ± 3.22 (standard error) in Xinjiang and −4.83 ± 3.30 in Jiangsu for micronaire in cotton, and −3.46 ± 1.03 for length of main raceme in rapeseed; and the corresponding *t* statistics (probabilities) were −2.19 (0.029), −1.77 (0.0789), and −4.31 (2.53E-05), respectively. Thus, population structure should be considered in real data analyses.

#### Association studies

Q matrix for population structure was incorporated into the genetic model of epistatic association mapping. A total of 11, 7, 5 and 2 reliable QTL were identified for micronaire in cotton based on trait phenotype, GCA, SCA and MPH, respectively (Table 1). A total of 18, 16, 2 and 2 reliable QTL were identified for length of main raceme in rapeseed based on trait phenotype, GCA, SCA and MPH, respectively (Table 2). These QTL were detected in at least two instances, each with a different dependent variable. Clearly, all types of effects were detected from trait phenotype, additive and *aa* effects were identified from GCA, and dominance-related effects were found from SCA and MPH.

### Genetic components of GCA, SCA and MPH

**Figure 1:**
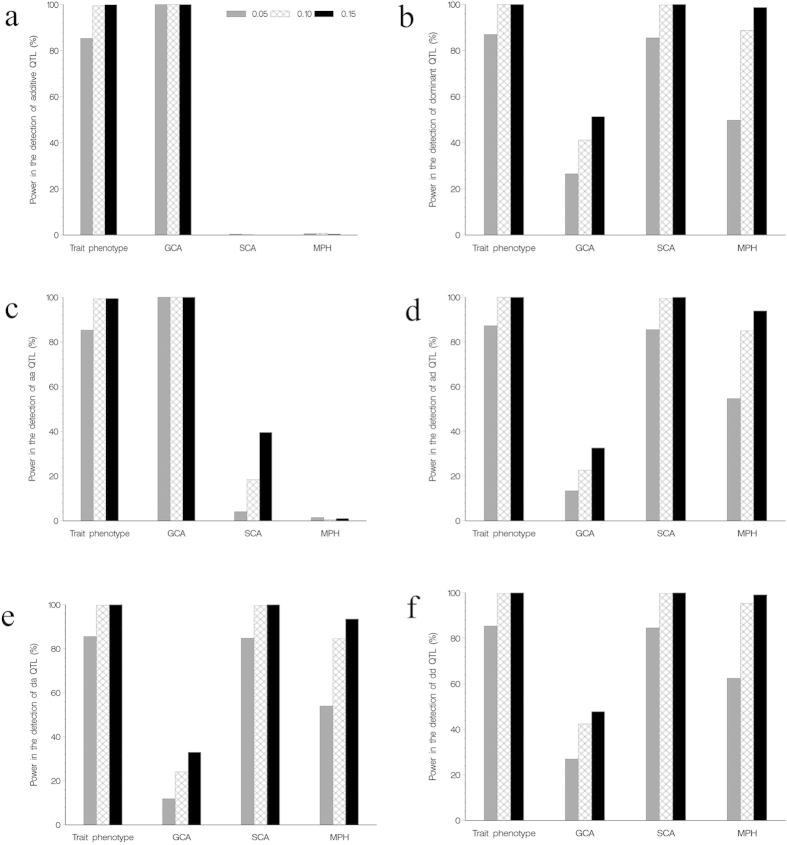
Power for detection of QTL in the genetic models of trait phenotype, general combining ability (GCA), specific combining ability (SCA) and mid-parental heterosis (MPH) in the NCII mating design. The gray, mesh and black bars represent the power of detecting QTL with a heritability of 0.05, 0.10 and 0.15, respectively.

In the model (2), trait phenotype, GCA, SCA and MPH were used as dependent variables. When all the simulated QTL had only one type of genetic effect in each experiment, the additive QTL was detected with dependent variables of trait phenotype and GCA but not of SCA and MPH (Fig. 1a). The additive effect is a component of GCA. A similar result was obtained in the association mapping for length of main raceme in rapeseed, because a total of 15 common additive QTL were detected when trait phenotype and GCA were used as dependent variable. Furthermore, one additional additive-by-additive × environment interaction for micronaire in cotton was detected from GCA but not from trait phenotype; and the *aa* QTL were more likely detected from GCA than from trait phenotype.

A dominant QTL could be detected with trait phenotype, SCA and MPH (Fig. 1b). The dominant effect is a component of SCA and MPH. Two common dominant QTL between trait phenotype and SCA and one common dominant QTL between SCA and MPH for length of main raceme in rapeseed supported this result, indicating that the power of detecting QTL was slightly higher for the trait phenotype and SCA models than for the MPH model (Fig. 1b). Although the dominant QTL could sometimes be identified in the GCA model, their estimated effect was close to zero (Table S2).

Although the *aa* QTL were detected in the model with trait phenotype, GCA and, sometimes, SCA models as dependent variable, the detection power were significantly higher with the trait phenotype and GCA than with the SCA and MPH. For example, the power in the detection of QTL with the 0.05 heritability was 100% using GCA, but the power in the detection of all the simulated QTL was less than 10% using MPH (Fig. 1c and Table S2). The *aa* effect is a key component of GCA. One similar *aa* QTL in the models with trait phenotype and GCA for length of main raceme in rapeseed validated this result (Table 2).

The *ad* QTL could be detected from trait phenotype, SCA, MPH and, sometimes, GCA models (Fig. 1d). Trait phenotype and SCA models had the highest and the GCA had the lowest power. Compared with the trait phenotype and SCA models, the power of MPH was relatively low because some of the *ad* QTL was identified as an additive QTL at marker positions 20, 40, 75, 90, 155 and 180 cM (Table S2), indicating that sometimes the *ad* QTL could not be distinguished from the additive QTL. Although sometimes the *ad* QTL in GCA model could be detected, its effect estimate was close to zero (Table S2). Similar results were found for the *da* and *dd* QTL, except that the *dd* QTL could be distinguished from the additive or dominant QTL with the MPH (Fig. 1e,f and Table S2). For association mapping of micronaire in cotton, two *ad* QTL and three *dd* QTL were identified with trait phenotype and SCA; these *ad* and *dd* effects were components of SCA. One *ad* QTL and one *dd* QTL were also detected by MPH, indicating that *ad* or *dd* QTL were less likely to be detected with MPH than with trait phenotype and SCA.

The above results showed that the additive and *aa* effects were the major contributors to GCA; some other effects, except the additive effect, were components of SCA, and the dominant-related effects were components of the MPH but a part of the *ad* or *da* QTL cannot be distinguished from the additive QTL.

### Relative contribution of genetic components to heterosis

**Figure 2:**
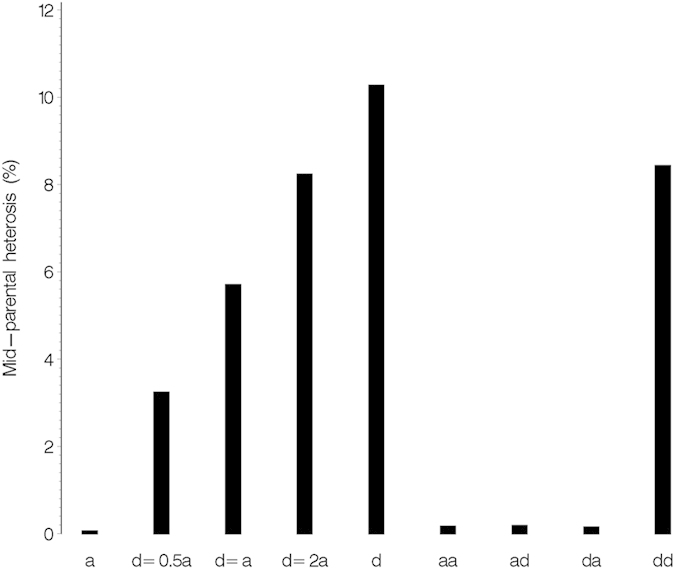
Average mid-parental heterosis in the 2,160,000 simulated F_1_ hybrids under the genetic models of additive (a), partial dominant (*d* = 0.5*a*), complete dominant (*d* = *a*), over-dominant (*d* = 2*a*), dominant (*a* = 0, *d*), additive-by-additive (*aa*), additive-by-dominant (*ad*), dominant-by-additive (*da*) and dominant-by-dominant (*dd*) QTL.

To further evaluate the genetic foundation of heterosis, we carried out three additional simulation experiments. In these three experiments, partial (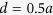), complete (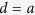) and over (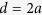) dominances were simulated, while the other parameters were the same as those in the first simulation experiment. In the three experiments, the powers of the dominant QTL detection with SCA and MPH increased as the degree of dominance increased (Table S3). When the above 2,160,000 simulated F_1_ hybrids, along with their parents, were used to calculate MPH, the absolute estimates of MPH under the dominance, *dd*, over-dominance, complete dominance and partial dominance genetic models were 10.29, 8.45, 8.25, 5.72 and 3.25 (%), respectively (Fig. 2), indicating that the magnitude of heterosis derived from the same set of QTL was dominance > dominance-by-dominance > over-dominance > complete dominance (Table S4).

### Effect of F_1_ hybrid proportion in NCII on association mapping

**Figure 3:**
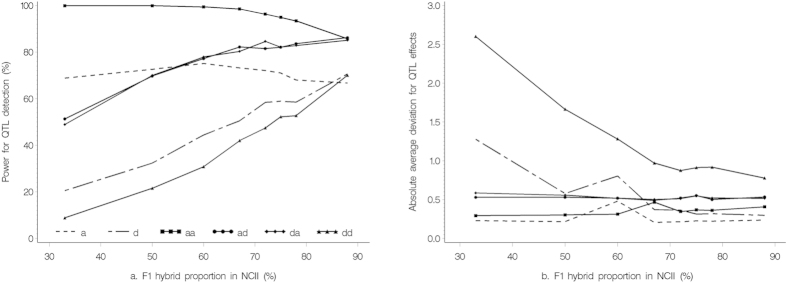
The effect of F_1_ hybrid proportion in the total number of maternal lines, paternal lines and F_1_ hybrids in the NCII (x-axis) on power of QTL detection (a) and absolute average deviation for QTL effect (b).

To investigate the effect of the mating design on association mapping, each maternal line was crossed with 1, 2, 3, 4, 5, 6, 7, and 15 paternal lines, the proportion of F_1_ hybrids in the total number of parental lines and F_1_ hybrids in the mapping population increased from 33% to 88% (Fig. 3 and S1). We found that the power of QTL detection slightly decreased for the additive and *aa* QTL, but significantly increased for the dominant-related QTL as the proportion of F_1_ hybrids in the mapping population increased, and the power was higher for the additive-related QTL than for the dominant and *dd* QTL (Fig. 3a). The decreases for the additive and *aa* QTL detection powers were due to the decrease of homozygotes in the mapping population. The absolute deviation slightly decreased for the additive-related effects, but significantly decreased for the dominant and *dd* effects as the proportion of F_1_ hybrids in the mapping population increased (Fig. 3b).

## Discussion

The current study is unique as compared to previous studies in the genetic dissection of heterosis. We assessed the relative importance of various genetic components of heterosis using a series of Monte Carlo simulation experiments and found that the ranking of factors affecting heterosis based on the same set of QTL was dominance > dominance-by-dominance > over-dominance > complete dominance. We used the F_1_ hybrids in the NCII mating design instead of bi-parental segregating populations to dissect the genetic foundation of heterosis, and identified different types of QTL contributing to trait phenotype, GCA, SCA and MPH. In this study, we also adopted a new QTL mapping model; for example, all the main and epistatic effects were included in one genetic model, which overcame the effect of background QTL on association mapping. Generally, the EBLASSO algorithm can estimate 100,000 effects in a sample of size 200. However, if the effect is too small or two QTL are closely linked, the power of association mapping is low as well; in this case, the empirical Bayesian elastic net^40^ is recommended.

GCA generally consists of additive and *aa* effects, and SCA consists of dominance-related effects. When considering GCA, our conclusion is consistent with previous reports because the additive and *aa* effects were correctly estimated in our model. However, the dominance and *dd* effect was not detected with GCA (Table S2), because the design matrix for the two genetic components was the same among different individuals. The same scenario was observed in Table S3. Although the other two dominance-related components can sometimes be detected in the genetic model of GCA, their estimates were close to zero, indicating that GCA was hardly associated with heterosis. This study observed that the *aa* effect was the smallest genetic component in the SCA model (Table S2), and a similar result was also reported by Bhullar *et al.*^41^, Singh *et al.*^42^, Cho and Scott^43^, Qi *et al.*^36^ and Qu *et al.*^34^. In the MPH model, *ad* or *da* QTL were partly identified as additive QTL so that the power in detecting the *ad* and *da* QTL was lower than those of other interactions (Table S2). Although trait phenotype was the best variable in the genetic dissection of quantitative traits or heterosis, other variables were beneficial to estimate some effects, e.g. trait phenotype and GCA were recommended for detecting additive and *aa* interaction effects, and trait phenotype and SCA for detecting dominance-related effects.

The NCII design is the most efficient genetic mating design for the analysis of combining ability^44^ and has been widely adopted in maize, rice and rapeseed breeding. In the genetic dissection of quantitative traits, the base population in NCII is often a bi-parental segregating population, such as BC_1_F_8_^34^, recombinant inbred lines^35^ and introgression lines^36^,^37^. In crop breeding, however, an elite F_1_ hybrid (high heterosis) is generally derived from the crosses between two kinds of inbred lines in maize breeding, and between sterile and restorer lines in rice and rapeseed breeding. This is why we imitate one hybrid breeding experiment in rapeseed in this study. Note that it is generally impractical to conduct all the possible crosses between base population (a series of sterile lines) and testers; thus, only a limited numbers of crosses are evaluated in field experiments. To be consistent with real crop breeding programs, a portion of the NCII populations was used for analysis in this study. By comparing the results from different mating strategies, we suggested that F_1_ hybrids and their parents be used if main-effect QTL need to be identified, but only F_1_ hybrids are required if epistatic QTL need to be identified.

In bi-parental segregating populations, such as F_2_, no significant differences in the estimates of positions, effects and detection powers of QTL were found between the models with trait phenotype and MPH (Table S5), as MPH is a linear function of the F_1_ trait phenotype. This result may be applicable to backcross, doubled haploid and recombinant inbred line populations.

The GCA model had higher power than the trait phenotype model in detecting additive and *aa* QTL (Fig. 1), which is confirmed by real data analysis in cotton, that is, A4-1 and A5-1 additive QTL detected by GCA are not detected by trait phenotype. SCA and trait phenotype had similar power in detecting dominant and *dd* QTL. SCA had lower power than trait phenotype, and MPH had slightly lower power than SCA in detecting *ad* and *da* QTL (Fig. S2 and Table S6). The proposed method provides choices in the dissection of genetic components of heterosis, and might be used further to validate the results (Tables 1 and 2). More importantly, mating design was often adopted in crop breeding, and the results we obtained from mating design could direct crop breeding.

Although a large population is recommended in current QTL mapping, sometimes a small population in crop breeding is also used to identify QTL^45^. Cui *et al.*^45^ found that a small breeding population with phenotypic selection has a high power to detect QTL. The cotton population in this study is a breeding population. In this population, each line of the eight parents is a chromosome segment substitution line with novel allele of various micronaire QTL. This is why the apparently good results are obtained in the small cotton population in this study.

## Conclusion

Main components are the additive and *aa* effects for GCA, and dominance-related effects for SCA and MPH. The *aa* interaction is a small component of SCA. The *ad* or *da* interaction for MPH is partly identified as an additive effect. The real datasets from rapeseed and cotton validated our findings. The ranking of genetic components that contribute to heterosis is dominance > dominance-by-dominance > over-dominance > complete dominance. In addition, if we increase the proportion of F_1_ hybrids in a partial NCII design, the power to detect dominance-related effects could be significantly increased, and the power to detect additive and *aa* effects could be slightly reduced.

## Methods

### NCII mating design in Monte Carlo simulation experiments

A random set of *a* cultivars as maternal lines was crossed with a random set of *b* cultivars as paternal lines to produce 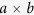 F_1_ hybrid combinations. When only a subset of the 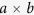 F_1_ hybrids was analyzed, we called this a partial NCII design. In the simulation study, we imitated one hybrid breeding experiment in rapeseed, in which each maternal line (sterile line) was crossed with two paternal lines (restorer lines); thus, the subset in this study was 2*a* F_1_ hybrids.

### Statistical model

#### Genetic model

The dependent variable *y*_*i*_ for the *i*th F_1_ hybrid in the NCII population can be described as


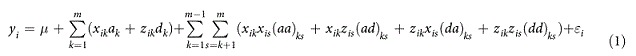


where four variables are considered separately as dependent variable, being trait phenotype, GCA, SCA and MPH; *μ* is the total average; *a*_*k*_ and *d*_*k*_ are additive and dominant effects of the *k*th QTL, respectively; 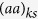, 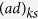, 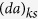 and 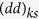 are *aa*, *ad*, *da* and *dd* interaction effects between the *k*th and *s*th QTL, respectively; *m* is the number of the putative QTL and each marker is resided by one putative QTL; 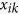 and 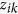 are dummy variables defined as 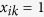 and 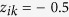 for the *k*th QTL genotype *QQ* of the *i*th individual, 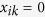 and 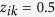 for *Qq*, and 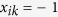 and 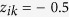 for *qq*; and 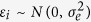 is the normally distributed random error.

In order to simplify the model (1), we rewrote the model (1) into the following matrix form


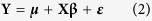


where 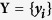, 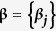 is the vector of the main and epistatic effects of QTL and **X** is the design matrix of all the QTL effects.

### Dependent variables in genetic model

In the genetic model (1) or (2), trait phenotype, GCA, SCA and MPH are dependent variable 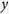. GCA (*g*_*i*_) is the mean performance of the *i*th parent in all its crosses with other parents, and SCA (*s*_*ij*_) between the *i*th and *j*th parents is the performance of their F_1_ hybrid measured as the deviation from the total expected GCA of the two parents. They are described as follows:


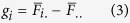



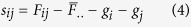


where *F*_*ij*_ is the phenotypic value of F_1_ hybrid between the *i*th and *j*th parents (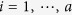;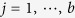); 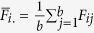 , and 
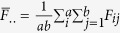
 . 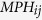 (%) refers to the superior performance (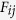) of the F_1_ hybrid relative to the average (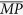) of the parental lines *i* and *j* and can be calculated as


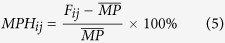


### Parameter estimation

Several methods could be applied to estimate the parameters in the model (1) or (2), such as penalized maximum likelihood^46^, Bayesian LASSO^47^,^48^, hierarchical generalized linear model^49^,^50^, empirical Bayes^51^ and EBLASSO^39^. Here, all the parameters were estimated using EBLASSO. We provide the main outline here; more details on the EBLASSO can be found in the study by Cai *et al.*^39^.

Three-level hierarchical prior distributions were employed in the EBLASSO. In the first level, *β*_*j*_ was set up to have an independent normal distribution with a mean of zero and unknown variance 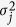. In the second level, 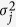 followed an independent exponential distribution with a common parameter λ: 
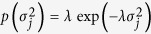
. In the third level, a conjugate Gamma prior distribution, Gamma (*a*, *b*), was used for the parameter *λ*. In this study, *a* and *b* were determined by three-fold cross-validation. In addition, non-informative uniform priors were used for *μ* and 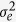. The major steps for the algorithm are as follows:

First, 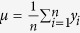 and 
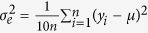
. Let 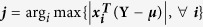, so 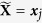. Let 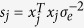 and 
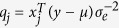
, so 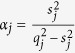. If 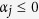, let 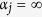.

The second step is the inner iteration. In this step, the purpose is to obtain a new 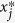. Let 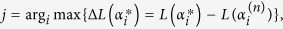 where 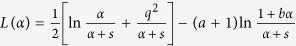; the new candidates 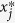 and 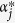 can be obtained. Three criteria related to *α*_*j*_ and 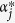 were used to determine whether 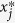 is to be retained in model (6):


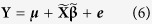


If 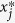 is retained in model (6), 
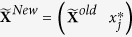
. Note that *μ* and 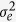 are fixed as constants. However, *s*_*j*_ and *q*_*j*_ need to be updated. If 
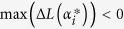
 or 
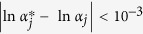
, the inner iteration converges, and 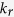 is obtained.

The third step is the outer iteration, and its purpose is to estimate 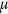 and 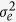 as shown below:


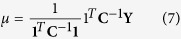



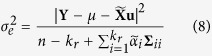


where 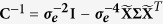, 
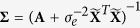
 (the covariance of **β**), **A**= 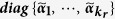, and 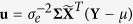 (empirical Bayes estimate of **β**). The outer iteration converges when the two conditions are simultaneously satisfied.

### Hypothesis test

The EBLASSO algorithm was used to select important effects from a full genetic model. When one effect was selected, its P-value in the *t*-test was provided as well. Here 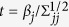, where 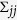 is the *j*th diagonal element of **Σ**. The probability threshold for declaring a significant main or epistatic effect was 0.05.

## Data Analyses

### Monte Carlo simulations

The purposes of the Monte Carlo simulation study were to compare four dependent variables in the genetic dissection of heterosis, to identify important components of heterosis and to investigate the effect of mating strategy on association mapping.

To compare four dependent variables in the genetic dissection of heterosis, six experiments were simulated (Table S1). In each experiment, 120 maternal lines, 120 paternal lines, and all the 120 × 120. F_1_ hybrids were simulated so that the GCA, SCA and MPH could be calculated. In the simulation with GCA as a dependent variable, all 240 parents were included in the mapping population. In the simulation study with trait phenotype, SCA and MPH as dependent variables, we created one hybrid breeding experiment in rapeseed with each maternal line (sterile line) crossed with two paternal lines (restorer lines), thus, a total of 240 F_1_ hybrids were generated and viewed as a mapping population. We simulated the mapping population and genotype using the method described by Lü *et al.*^52^. Sixty equally spaced markers, each with two alleles of equal proportions, were simulated on three chromosome segments; the length of each segment was 95 cM. The genotypes of all the F_1_ hybrids were then deduced from the simulated parental genotypes. In each experiment, the simulated data had six QTL: two each at 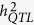
= 0.05, 0.10, and 0.15, and each QTL had two alleles of the same frequency. Based on these heritabilities and residual variance 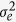, the total genetic variance 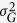 was estimated by 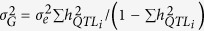, which was further partitioned into each QTL. The QTL effect was determined by its genetic variance and allelic frequency. Six QTL with main effects in the first and second experiments were placed on marker positions 25 (chr. 1), 75 (chr. 1), 135 (chr. 2), 175 (chr. 2), 220 (chr. 3) and 270 cM (chr. 3), respectively; and six epistatic QTL in the third to sixth experiments were located on marker pairs at 20 & 60, 90 & 125, 155 & 205, 180 & 235, 40 & 275 and 75 & 220 cM, respectively. One type of QTL effect was assigned to all the six QTL in each experiment so that additive, dominant, *aa*, *ad*, *da* and *dd* effects were assigned to the first to sixth experiments, respectively (Table S1). Each simulation consisted of 1,000 replications. For each simulated QTL, we counted the number of samples in which the P-value < 0.05 and its ratio to the total number of replications (1,000) to represent the empirical power of this QTL.

To identify important components of heterosis, three additional experiments with partial (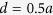), complete (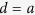) and over (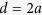) dominances of QTL were conducted. The other parameters in the three experiments were similar to those used in the cases 1~6 listed in Table S1. In these nine experiments, all the F_1_ individuals along with their parents were used to calculate the MPH and the relative sizes of MPH were used to measure the contribution of the genetic components to heterosis.

To investigate the effect of mating strategy on QTL mapping, eight simulation experiments were carried out by allowing one maternal line to be crossed with 1, 2, 3, 4, 5, 6, 7 and 15 paternal lines. To ensure a stable sample size, the mapping populations in the eight experiments were 80 (maternal) + 80 (F_1_, the *i*th maternal line (*M*_*i*_) × the *i*th paternal line (*P*_*i*_), 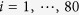) + 80 (paternal), 60 (maternal) + 60 × 2 (F_1_: *M*_*i*_×*P*_*i*_ and 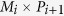, 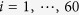. If 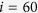, 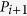 was changed into *P*_59_) + 60 (paternal), 48 (maternal) + 48 × 3 (F_1_) + 48 (paternal), 40 (maternal) + 40 × 4 (F_1_) + 40 (paternal), 34 (maternal) + (34 × 5 + 2) (F_1_: the additional 2 F_1_ hybrids were 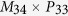 and 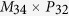) + 34 (paternal), 30 (maternal) + 30 × 6 (F_1_) + 30 (paternal), 26 (maternal) + (26 × 7 + 6) (F_1_: the additional 6 F_1_ hybrids were from 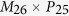 to *M*_26_ × *P*_20_) + 26 (paternal), and 15 (maternal) + 15 × 15 (F_1_) + 15 (paternal), respectively (Fig. S1). For the efficiency of simulation, twenty-one equally spaced markers, each with two alleles of equal frequency, were simulated on one chromosome with a total length of 100 cM. In each experiment, six QTL with a heritability of 0.05 were simulated; and each QTL locus had only one type of effect. An additive (dominant) QTL was located at marker position 20 (85) cM; the *aa*, *ad*, *da* and *dd* interaction QTL were located between marker pairs 10 & 30, 40 & 55, 45 & 80 and 65 & 95 cM, respectively. The other parameters were the same as those in the first simulation experiment (Table S1).

### Real datasets analyzed

A cotton dataset provided by Dr. Tianzhen Zhang’s group at Nanjing Agricultural University, China was used for the demonstration. The dataset contained phenotypes of micronaire (a fibre characteristic) from 8 parents and their 28 F_1_ hybrids which were grown at two locations: Xinjiang and Jiangsu provinces, China. All the eight parents were chromosome segment substitution lines, and bred from the crosses of TM-1 and cultivars with novel alleles of various micronaire QTL. Among these parents, there were fifteen chromosome substituted segments, which were located on 9 chromosomes and identified by 15 SSR markers. In the genetic model, 30 main effects, one environmental effect, 420 epistasis effects, 30 QTL-by-environment effects and 420 epistasis-by-environment effects were considered.

A rapeseed (*Brassica napus*) dataset provided by Dr. Jinxing Tu’s group at Huazhong Agricultural University, China was also used for the further demonstration. The data for length of main raceme were collected from 298 sterile lines, 143 restorer lines (restoring fertility of the F_1_ hybrid from male sterile line) and 284 F_1_ hybrids at Huazhong Agricultural University in 2010. A total of 205 SSR primer pairs were used to screen for polymorphisms among all the 441 parents and the genotypes of all the F_1_ hybrids were deduced from their parents. The total number of effects included in the genetic model is 84050.

All the parameters were estimated by EBLASSO^39^. In real data analyses, the best estimates for parameters *a* and *b* in the Gamma (*a*, *b*) distribution were determined from three-, five- and ten-fold cross-validations. The software (GAS_NCII) is available. The critical value of the P-value for statistical significance was set to 0.05. Q matrix was calculated using Structure 2.3.4 (http://pritchardlab.stanford.edu/structure.html), and incorporated into the genetic model of association mapping in real data analysis.

### LD score regression

Bulik-Sullivan *et al.*^53^ proposed linkage disequilibrium (LD) score regression to distinguish between inflation from a true polygenic signal and population stratification bias for a binary trait. In the regression of 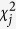 between the *j*th marker and binary trait on LD score 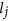 (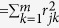,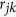 is the correlation coefficient between the *j*th and *k*th markers, and *m* is the number of markers), significant difference between the regression intercept estimate and one indicates the significant effect of population structure on association mapping. If the trait under consideration is continuous, extremely large (35%) and small (35%) values are transferred into 1 and 0 (binary), respectively, and only 70% of individuals are adopted in the LD score regression.

## Additional Information

**How to cite this article**: Wen, J. *et al.* Genetic dissection of heterosis using epistatic association mapping in a partial NCII mating design. *Sci. Rep.* **5**, 18376; doi: 10.1038/srep18376 (2015).

## Supplementary Material

Supplementary Information

## Figures and Tables

**Table 1:** Position, type and effect of QTL for cotton micronaire in a mating design.

QTL		Trait phenotype			General combining ability			Specific combining ability			Mid-parental heterosis		
Position	Type	Effect	P-value	r^2^ (%)	Effect	P-value	r^2^(%)	Effect	P-value	r^2^(%)	Effect	P-value	r^2^(%)
A1-1	*a*	0.22	1.12E-05	7.33	0.09	4.89E-13	15.87						
A4-1	*a*	−0.08	0.04	1.08	−0.04	1.47E-08	3.00						
A5-1	*a*	−0.33	1.14E-06	11.48	−0.17	<1E-300	50.56						
A5-2	*a*	0.12	0.0070	0.50	0.06	8.54E-11	7.98						
A13-2	*a*	0.25	1.06E-06	8.06	0.10	6.51E-14	20.73						
A4-1×A13-2	*ad*	0.35	1.67E-08	10.53				0.35	8.17E-08	13.73			
A4-1×D2-1	*ad*	−0.30	3.79E-08	9.00				−0.32	4.26E-08	15.25			
A5-2×D2-1	*da*	0.34	1.38E-09	8.36	−0.01 (aa*E)	5.10E-03	0.48				5.46	0.0002	17.72
A5-1×A13-2	*dd*	0.31	0.0004	4.07				0.33	0.0001	7.19			
A5-1×D2-1	*dd*	−0.26	0.0015	1.79				−0.21	0.0048	3.29			
A5-2×A13-2	*dd*	0.75	1.05E-12	16.79				0.70	5.56E-11	28.32	10.32	8.71E-05	27.70
A1-1*A11-1	aa*E				0.01	5.93E-03	0.19						

*a*: additive; *ad*: additive × dominance; *da*: dominance × additive; *dd*: dominance × dominance; aa*E: interaction between additive-by-additive effect and environment.

**Table 2:** Position, type and effect of QTL for rapeseed length of main raceme in a partial NCII mating design.

QTL		Trait phenotype			General combining ability			Specific combining ability			Mid-parental heterosis		
Position	Type	Effect	P-value	r^2^(%)	Effect	P-value	r^2^(%)	Effect	P-value	r^2^(%)	Effect	P-value	r^2^(%)
CN75b	*a*	−1.30	8.84E-05	1.70	−0.55	<1E-300	2.38						
**BRMS-036a**	*a*	−1.37	1.07E-06	2.90	−0.56	<1E-300	4.00						
**BRMS-036c**	*a*	−1.14	0.0002	1.65	−0.53	<1E-300	2.81						
Ol11-B05b	*a*	1.58	1.34E-06	3.34	−0.15	9.11E-05	0.19				−0.02	0.0004	1.84
xy2b	*a*	1.00	0.0009	1.45	0.24	1.56E-09	0.54						
20-1c	*a*	0.69	0.0060	0.81	0.18	1.80E-07	0.41						
Bn1b	*a*	0.63	0.0111	0.57	0.46	<1E-300	2.44						
Ol12-E03B	*a*	−0.62	0.0091	0.71	−0.25	3.53E-12	0.79						
CB10036B	*a*	−1.11	0.0012	0.94	−0.70	<1E-300	3.39						
Na10-F08	*a*	−1.49	0.0006	1.18	−1.11	<1E-300	4.39						
Na12-A02A	*a*	1.81	2.02E-05	1.88	1.14	<1E-300	5.86						
Na12-A02B	*a*	−0.65	0.0075	0.51	−0.37	<1E-300	1.78						
BnGMS352A	*a*	−0.95	0.0006	1.23	−0.64	<1E-300	4.69						
Na10-C06A	*a*	0.60	0.0109	0.78	0.30	2.28E-13	1.21						
Na14-H11A	*a*	−0.79	0.0022	0.68	−0.44	<1E-300	2.19						
Na12-A02C	*d*	2.24	0.0036	0.97				1.79	1.53E-12	14.89	0.05	9.20E-10	7.59
BnGMS385C	*d*	2.61	0.0012	1.30				2.03	4.23E-12	14.85			
CN46d × Ra3-E05C	*aa*	−0.99	2.88E-05	2.18									
CN46b × Ra3-E05C	*aa*				0.01	0.0020	4.81E-04						

*a*: additive; *d*: dominance; *aa*: additive × additive. Markers with bold-type letter were associated with length of main raceme in rapeseed in previous studies.
