# Supplementary material for: Genetic dissection of heterosis using epistatic association mapping in a partial NCII mating design
Source: Sci Rep. 2015 Dec 17;5:18376. doi: 10.1038/srep18376 (PMC4683666; doi:10.1038/srep18376)
Supplement: Supplementary Information [file srep18376-s1.pdf]

Genetic dissection of heterosis using epistatic association mapping in a partial NCII mating design

Jia Wen[1,2], Xinwang Zhao[1], Guorong Wu[2], Dan Xiang[2], Qing Liu[2], Su-Hong Bu[2], Can Yi[2], Qijian Song[3], Jim M. Dunwell[4], Jinxing Tu[1], Tianzhen Zhang[2] & Yuan-Ming Zhang[1]

Table S1 | Simulated parameters in all the simulation experiments

Simulation study	Case	NCII design			Genome length (cM)	Marker Density (cM)	QTL	Effect type of QTL	Heritability (%)	Position(cM)	Sample for analysis			
		maternal	paternal	F_1							Trait phenotype	GCA	SCA	MPH
Part I	1	120	120	240	100	Equal, 5	1	*a*	5	25	240 F_1	240 parents	240 F_1	240 F_1
							2	*a*	10	75				
							3	*a*	5	135				
							4	*a*	15	175				
							5	*a*	10	220				
							6	*a*	15	270				
	2	120	120	240	100	Equal, 5	1	*d*	5	25	240 F_1	240 parents	240 F_1	240 F_1
							2	*d*	10	75				
							3	*d*	5	135				
							4	*d*	15	175				
							5	*d*	10	220				
							6	*d*	15	270				
	3	120	120	240	100	Equal, 5	1	*aa*	5	20 × 60	240 F_1	240 parents	240 F_1	240 F_1
							2	*aa*	10	90 × 125				
							3	*aa*	5	155 × 205				
							4	*aa*	15	180 × 235				
							5	*aa*	10	40 × 275				
							6	*aa*	15	75 × 220				
	4	120	120	240	100	Equal, 5	1	*ad*	5	20 × 60	240 F_1	240 parents	240 F_1	240 F_1
							2	*ad*	10	90 × 125				
							3	*ad*	5	155 × 205				
							4	*ad*	15	180 × 235				
							5	*ad*	10	40 × 275				
							6	*ad*	15	75 × 220				
	5	120	120	240	100	Equal, 5	1	*da*	5	20 × 60	240 F_1	240 parents	240 F_1	240 F_1
							2	*da*	10	90 × 125				
							3	*da*	5	155 × 205				
							4	*da*	15	180 × 235				
							5	*da*	10	40 × 275				
							6	*da*	15	75 × 220				
	6	120	120	240	100	Equal, 5	1	*dd*	5	20 × 60	240 F_1	240 parents	240 F_1	240 F_1
							2	*dd*	10	90 × 125				
							3	*dd*	5	155 × 205				

							4	*dd*	15	180 × 235				
							5	*dd*	10	40 × 275				
							6	*dd*	15	75 × 220				
Part II	7	120	120	240	100	Equal, 5	1	d=0.5a	5	25				
							2	d=0.5a	10	75				
							3	d=0.5a	5	135	240 F_1	240 parents	240 F_1	240 F_1
							4	d=0.5a	15	175				
							5	d=0.5a	10	220				
							6	d=0.5a	15	270				
	8	120	120	240	100	Equal, 5	1	d=a	5	25				
							2	d=a	10	75				
							3	d=a	5	135	240 F_1	240 parents	240 F_1	240 F_1
							4	d=a	15	175				
							5	d=a	10	220				
							6	d=a	15	270				
	9	120	120	240	100	Equal, 5	1	d=2a	5	25				
							2	d=2a	10	75				
							3	d=2a	5	135	240 F_1	240 parents	240 F_1	240 F_1
							4	d=2a	15	175				
							5	d=2a	10	220				
							6	d=2a	15	270				
Part III	10	80	80	80	100	Equal, 5	1	*a*	5	20				
							2	*d*	5	85				
							3	*aa*	5	10 × 30	160 parents+ 80 F_1			
							4	*ad*	5	40 × 55				
							5	*da*	5	45 × 80				
							6	*dd*	5	65 × 95				
	11	60	60	120	100	Equal, 5	1	*a*	5	20				
							2	*d*	5	85				
							3	*aa*	5	10 × 30	120 parents+ 120 F_1			
							4	*ad*	5	40 × 55				
							5	*da*	5	45 × 80				
							6	*dd*	5	65 × 95				
	12	48	48	144	100	Equal, 5	1	*a*	5	20				
							2	*d*	5	85				
							3	*aa*	5	10 × 30	96 parents+ 144 F_1			
							4	*ad*	5	40 × 55				
							5	*da*	5	45 × 80				

						6	*dd*	5	65 × 95	
13	40	40	160	100	Equal, 5	1	*a*	5	20	
						2	*d*	5	85	
						3	*aa*	5	10 × 30	80 parents+ 160 F_1
						4	*ad*	5	40 × 55	
						5	*da*	5	45 × 80	
						6	*dd*	5	65 × 95	
14	34	34	172	100	Equal, 5	1	*a*	5	20	
						2	*d*	5	85	
						3	*aa*	5	10 × 30	68 parents+ 172 F_1
						4	*ad*	5	40 × 55	
						5	*da*	5	45 × 80	
						6	*dd*	5	65 × 95	
15	30	30	180	100	Equal, 5	1	*a*	5	20	
						2	*d*	5	85	
						3	*aa*	5	10 × 30	60 parents+ 180 F_1
						4	*ad*	5	40 × 55	
						5	*da*	5	45 × 80	
						6	*dd*	5	65 × 95	
16	26	26	188	100	Equal, 5	1	*a*	5	20	
						2	*d*	5	85	
						3	*aa*	5	10 × 30	52 parents+ 188 F_1
						4	*ad*	5	40 × 55	
						5	*da*	5	45 × 80	
						6	*dd*	5	65 × 95	
17	15	15	225	100	Equal, 5	1	*a*	5	20	
						2	*d*	5	85	
						3	*aa*	5	10 × 30	30 parents+ 225 F_1
						4	*ad*	5	40 × 55	
						5	*da*	5	45 × 80	
						6	*dd*	5	65 × 95	

Table S2 | Comparisons of trait phenotype, general combining ability (GCA), specific combining ability (SCA) and mid-parental heterosis (MPH) in the genetic dissection of heterosis under additive, dominance, *aa*, *ad*, *da* and *dd* genetic models using Monte Carlo simulation experiments

QTL	True value				Estimate																	
					Trait phenotype									GCA								
	Posi (cM)		Effect	r^2	Posi (cM)		*a*	*d*	*aa*	*ad*	*da*	*dd*	Power(%)	Posi (cM)		*a*	*d*	*aa*	*ad*	*da*	*dd*	Power(%)
a	25		1.5811	0.05	24.99<br>(0.34)		1.6386<br>(0.4046)	-0.0189<br>(0.4530)					85.0	25.00<br>(0.000		0.7744<br>(0.0592)	-0.0001<br>(0.0349)					100.0
	75		2.2361	0.10	75.00<br>(0.00)		2.2179<br>0.3593	-0.0104<br>0.4303					99.5	75.00<br>(0.00)		1.0917<br>(0.0806)	-0.0001<br>(0.0349)					100.0
	135		1.5811	0.05	135.01<br>(0.17)		1.6444<br>(0.3250)	-0.0175<br>(0.4260)					85.7	135.00<br>(0.00)		0.7707<br>(0.0596)	-0.0001<br>(0.0349)					100.0
	175		2.7386	0.15	174.99<br>(0.16)		2.7093<br>(0.3594)	0.0135<br>(0.4181)					99.8	175.00<br>(0.00)		1.3406<br>(0.0855)	-0.0001<br>(0.0349)					100.0
	220		2.2361	0.10	219.97<br>(0.69)		2.2096<br>(0.4437)	-0.0059<br>(0.4475)					99.4	220.00<br>90.00)		1.0922<br>(0.0787)	-0.0001<br>(0.0349)					100.0
	270		2.7386	0.15	270.00<br>(0.06)		2.7117<br>(0.3871)	0.0147<br>(0.4641)					100.0	270.00<br>(0.00)		1.3363<br>(0.0915)	-0.0001<br>(0.0349)					100.0
d	25		2.2361	0.05	25.00<br>(0.07)		0.0096<br>(0.2978)	2.2850<br>(0.4158)					87.2	25.01<br>(0.66)		0.0095<br>(0.1252)	-0.0108<br>(0.0059)					26.9
	75		3.1623	0.10	75.00<br>(0.22)		0.0113<br>(0.2985)	3.1371<br>(0.4813)					99.9	75.00<br>(0.77)		-0.0076<br>(0.1563)	-0.0107<br>(0.0061)					42.3
	135		2.2361	0.05	135.00<br>(0.07)		0.0107<br>(0.3253)	2.3163<br>(0.3983)					86.6	134.87<br>(0.92)		-0.0038<br>(0.1264)	-0.0109<br>(0.0060)					26.1
	175		3.8730	0.15	175.00<br>(0.14)		-0.0005<br>(0.3411)	3.8639<br>(0.4689)					100.0	175.01<br>(0.24)		-0.0044<br>(0.1721)	-0.0107<br>(0.0062)					51.5
	220		3.1623	0.10	220.00<br>(0.01)		0.0005<br>(0.3885)	3.1456<br>(0.4544)					99.9	220.01<br>(0.25)		0.00005<br>(0.1498)	-0.0108<br>(0.0063)					40.0
	270		3.8730	0.15	270.02<br>(0.50)		0.0079<br>(0.3046)	3.8482<br>(0.4921)					100.0	269.96<br>(0.63)		0.0075<br>(0.1733)	-0.0108<br>(0.0064)					51.0
aa	20	60	2.2361	0.05	19.91<br>(2.59)	60.01<br>(0.22)			2.3059<br>(0.4438)	0.0001<br>(0.6016)	-0.0080<br>(0.5968)	0.0147<br>(0.8694)	85.4	19.99<br>(0.33)	60.00<br>(0.00)			0.5477<br>(0.0415)	-0.0009<br>(0.0686)	0.0014<br>(0.0698)	0.0001<br>(0.0178)	100.0
	90	125	3.1623	0.10	90.00<br>(0.87)	125.01<br>(0.35)			3.1540<br>(0.5016)	-0.0245<br>(0.6219)	-0.0145<br>(0.6010)	0.0093<br>(0.8707)	99.4	90.00<br>(0.07)	125.00<br>(0.03)			0.7780<br>(0.0443)	-0.0013<br>(0.0788)	-0.0031<br>(0.0796)	0.0001<br>(0.0178)	100.0
	155	205	2.2361	0.05	155.00<br>(0.00)	205.03<br>(0.84)			2.3237<br>(0.4190)	0.0139<br>(0.5825)	0.0090<br>(0.6114)	0.0115<br>(0.8336)	85.2	155.00<br>(0.01)	205.00<br>(0.03)			0.5498<br>(0.0319)	-0.0044<br>(0.0702)	-0.0030<br>(0.0709)	0.0001<br>(0.0178)	100.0
	180	235	3.8730	0.15	179.99<br>(0.46)	234.97<br>(0.67)			3.8299<br>(0.5308)	0.0150<br>(0.6070)	0.0022<br>(0.6013)	0.0020<br>(0.8225)	100.0	180.00<br>(0.04)	235.00<br>(0.05)			0.9514<br>(0.0538)	0.0009<br>(0.0787)	-0.0007<br>(0.0758)	0.0001<br>(0.0178)	100.0
	40	275	3.1623	0.10	40.00<br>(0.02)	274.93<br>(2.09)			3.1298<br>(0.5024)	-0.0411<br>(0.6111)	0.0064<br>(0.6223)	-0.0126<br>(0.8590)	99.1	40.00<br>(0.04)	275.00<br>(0.07)			0.7769<br>(0.0472	0.0011<br>(0.0767)	-0.0031<br>(0.0753)	0.0001<br>(0.0178)	100.0
	75	220	3.8730	0.15	74.99<br>(0.61)	220.01<br>(0.21)			3.8575<br>(0.5159)	0.0101<br>(0.5753)	0.0087<br>(0.6172)	-0.0055<br>(0.8637)	99.9	75.00<br>(0.06)	220.00<br>(0.05)			0.9522<br>(0.0543)	-0.0050<br>(0.0770)	0.0024<br>(0.0806)	0.0001<br>(0.0178)	100.0
ad	20	60	3.1623	0.05	20.07<br>(3.00)	60.00<br>(0.08)			-0.0124<br>(0.4217)	3.2726<br>(0.5721)	-0.0136<br>(0.6072)	0.0126<br>(0.8662)	86.1	19.92<br>(1.53)	60.19<br>(1.51)			0.0087<br>(0.1182)	-0.0154<br>(0.0489)	0.0086<br>(0.0771)	-0.0001<br>(0.0037)	12.9
	90	125	4.4721	0.10	89.97<br>(0.77)	124.99<br>(0.21)			0.0159<br>(0.4183)	4.4339<br>(0.6949)	-0.0177<br>(0.5989)	0.0349<br>(0.8546)	99.8	89.96<br>(0.66)	125.00<br>(0.93)			-0.0004<br>(0.1341)	-0.0206<br>(0.0515)	0.0046<br>(0.0826)	0.000003<br>(0.0038)	23.2
	155	205	3.1623	0.05	155.01<br>(0.51)	204.99<br>(0.16)			-0.0231<br>(0.4475)	3.2707<br>(0.6015)	0.0204<br>(0.5946)	-0.0071<br>(0.8541)	88.5	155.04<br>(0.42)	205.19<br>(1.39)			0.0035<br>(0.1143)	-0.0148<br>(0.0487)	-0.0047<br>(0.0738)	-0.0002<br>(0.0036)	13.9
	180	235	5.4772	0.15	180.02<br>(0.49)	235.01<br>(0.34)			-0.0049<br>(0.4535)	5.4358<br>(0.7158)	0.0136<br>(0.6194)	0.0151<br>(0.8421)	100.0	179.99<br>(0.67)	235.04<br>(0.68)			0.0066<br>(0.1472)	-0.0228<br>(0.0529)	-0.0041<br>(0.0808)	0.0001<br>(0.0039)	33.4

	40	275	4.4721	0.10	40.00	275.00	-0.0032	4.4584	-0.0228	-0.0270	99.9	40.02	274.93	0.0079	-0.0188	0.0028	-0.00002	22.0
					(0.01)	(0.07)	(0.4406)	(0.6939)	(0.6141)	(0.8724)		(0.89)	(0.72)	(0.1253)	(0.0447)	(0.0858)	(0.0039)	
	75	220	5.4772	0.15	75.00	220.02	0.0222	5.4846	-0.0172	0.0378	100.0	74.92	220.04	0.0132	-0.0183	0.0076	-0.0002	31.7
					(0.09)	(0.55)	(0.4497)	(0.7634)	(0.5926)	(0.8372)		(0.88)	(1.00)	(0.1464)	(0.0493)	(0.0816)	(0.0040)	
	20	60	3.1623	0.05	19.96	59.86	-0.0052	-0.0184	3.2686	-0.0221	85.8	20.37	60.17	-0.0032	0.0085	-0.0102	0.0002	11.9
					(1.21)	(3.92)	(0.4819)	(0.6150)	(0.6652)	(0.8392)		(2.84)	(4.39)	(0.1105)	(0.0806)	(0.0468)	(0.0038)	
	90	125	4.4721	0.10	90.00	124.91	0.0067	0.0101	4.4312	-0.0007	99.7	90.31	124.98	0.0078	-0.0056	-0.0171	0.0002	23.5
					(0.00)	(2.24)	(0.4562)	(0.5995)	(0.7386)	(0.8136)		(8.57)	(0.57)	(0.1247)	(0.0788)	(0.0500)	(0.0037)	
	155	205	3.1623	0.05	155.02	204.99	-0.0089	-0.0047	3.2314	-0.0059	85.4	155.04	204.83	-0.0022	-0.0143	-0.0103	0.0004	11.8
					(0.27)	(0.58)	(0.4576)	(0.6128)	(0.6205)	(0.8847)		(0.46)	(1.71)	(0.1108)	(0.0734)	(0.0462)	(0.0037)	
da	180	235	5.4772	0.15	179.98	234.88	-0.0067	0.0055	5.4453	0.0495	100.0	180.13	235.01	0.0116	-0.0053	-0.0195	0.0002	34.1
					(0.53)	(3.83)	(0.4559)	(0.6162)	(0.8243)	(0.8552)		(2.16)	(0.90)	(0.1422)	(0.0796)	(0.0503)	(0.0037)	
	40	275	4.4721	0.10	39.99	275.00	-0.0069	0.0197	4.4347	0.0110	99.8	39.96	275.06	0.0001	-0.0021	-0.0215	0.000008	24.7
					(1.29)	(0.34)	(0.4541)	(0.6136)	(0.7244)	(0.8279)		(0.64)	(0.84)	(0.1260)	(0.0724)	(0.0474)	(0.0037)	
	75	220	5.4772	0.15	75.00	220.01	0.0081	0.0098	5.4277	0.0388	100.0	74.96	220.02	0.0006	0.0033	-0.0168	0.0003	31.8
					(0.32)	(0.34)	(0.4607)	(0.6022)	(0.8416)	(0.8625)		(0.82)	(0.33)	(0.1464)	(0.0801)	(0.0473)	(0.0040)	
	20	60	4.4721	0.05	19.98	59.99	0.0110	0.0095	-0.0073	4.5834	86.0	19.92	59.94	0.0035	-0.0006	-0.0018	-0.0077	26.5
					(0.41)	(0.34)	(0.4179)	(0.5991)	(0.5998)	(0.8441)		(0.75)	(1.65)	(0.1271)	(0.0438)	(0.0482)	(0.0046)	
	90	125	6.3246	0.10	89.98	124.99	0.0100	0.0102	-0.0082	6.2686	99.8	90.00	124.98	-0.0023	0.0026	-0.0006	-0.0072	42.5
					(1.26)	(0.32)	(0.4351)	(0.6171)	(0.5954)	(0.9940)		(0.59)	(0.60)	(0.1512)	(0.0500)	(0.0470)	(0.0045)	
	155	205	4.4721	0.05	155.04	205.00	-0.0088	-0.0012	-0.0051	4.6106	88.2	155.00	205.07	-0.0075	-0.0005	-0.0002	-0.0079	27.4
					(1.18)	(0.04)	(0.4223)	(0.6051)	(0.5773)	(0.8283)		(0.86)	(1.97)	(0.1277)	(0.0482)	(0.0478)	(0.0048)	
dd	180	235	7.7460	0.15	180.00	235.01	-0.0029	-0.0017	0.0198	7.6976	100.0	180.00	235.00	0.0130	0.00001	0.0022	-0.0076	47.3
					(0.10)	(0.36)	(0.4341)	(0.6236)	(0.6123)	(1.0334)		(0.09)	(0.33)	(0.1705)	(0.0472)	(0.0465)	(0.0047)	
	40	275	6.3246	0.10	40.01	274.99	0.00002	0.0342	-0.0021	6.3201	99.6	40.01	275.04	-0.0088	0.0015	-0.0057	-0.0074	42.3
					(0.25)	(0.23)	(0.4429)	(0.6207)	(0.6138)	(0.9553)		(0.94)	(0.55)	(0.1520)	(0.0468)	(0.0464)	(0.0044)	
	75	220	7.7460	0.15	75.00	220.00	0.0090	-0.0296	0.0148	7.6949	100.0	75.03	219.92	0.0058	-0.0004	0.0021	-0.0073	48.2
					(0.15)	(0.13)	(0.4405)	(0.6035)	(0.6101)	(1.0650)		(0.50)	(1.13)	(0.1679)	(0.0486)	(0.0487)	(0.0048)	

Continue

QTL	True value			Estimate															
				SCA								MPH							
	Posi (cM)	Effect	r^2	Posi (cM)	*a*	*d*	*aa*	*ad*	*da*	*dd*	Power(%)	Posi (cM)	*a*	*d*	*aa*	*ad*	*da*	*dd*	Power(%)
a	25	1.5811	0.05	24.00 (8.22)	0.1288 (0.8916)	-0.1679 (1.2862)					0.5	27.50 (6.12)	-0.2192 (0.8502)	-0.2339 (1.5662)					0.6
	75	2.2361	0.10	75.00 (.)	1.0988 (.)	0.8017 (.)					0.1	75.00 (5.77)	0.4026 (0.7830)	0.0480 (1.6957)					0.7
	135	1.5811	0.05	142.50 (3.54)	-0.0281 (1.3959)	0.2919 (0.1842)					0.2	140.00 (8.66)	-0.5152 (1.2851)	-0.3542 (0.9427)					0.5
	175	2.7386	0.15	.	.	.					.	172.82 (6.80)	-0.1850 (0.8502)	-0.4381 (1.0790)					0.6
	220	2.2361	0.10	220.00 (14.14)	0.1915 (1.6123)	-0.0661 (0.1948)					0.2	217.14 (8.09)	0.4064 (1.0138)	-0.1195 (0.7870)					0.7
	270	2.7386	0.15	270.00 (.)	-0.3093 (.)	1.4785 (.)					0.1	270.00 (.)	-1.2453 (.)	0.3162 (.)					0.1
d	25	2.2361	0.05	25.00 (0.07)	0.0025 (0.3182)	2.2723 (0.3750)					85.3	24.97 (0.51)	0.0012 (0.4233)	2.7683 (0.4797)					50.3
	75	3.1623	0.10	75.01 (0.34)	0.0123 (0.3485)	3.1045 (0.4864)					99.7	75.00 (0.05)	0.0027 (0.4374)	3.5317 (0.6373)					88.2
	135	2.2361	0.05	135.00 (0.00)	-0.0049 (2.1594)	2.2958 (1.9086)					85.6	134.99 (0.34)	-0.0441 (0.4549)	2.7916 (0.5282)					49.3
	175	3.8730	0.15	175.00 (0.00)	-0.0026 (2.5693)	3.8265 (2.2354)					100.0	175.03 (1.13)	0.0026 (0.4389)	4.2471 (0.6523)					98.3
	220	3.1623	0.10	220.00 (0.03)	-0.0008 (0.3392)	3.1078 (0.4727)					99.7	220.00 (0.21)	0.0034 (0.4350)	3.5390 (0.6109)					89.1
	270	3.8730	0.15	220.00 (0.16)	-0.0022 (0.3860)	3.8265 (0.4919)					100.0	270.00 (0.16)	0.0171 (0.4383)	4.2314 (0.6691)					99.0
aa	20 60	2.2361	0.05	20.66 (2.37) 59.61 (1.79)			1.9478 (0.8433)	0.1087 (0.9676)	0.0018 (0.8876)	0.1252 (0.8191)	3.8	21.67 (6.99) 63.55 (8.58)			-0.1614 (1.6016)	0.8368 (1.8678)	-0.2007 (2.0537)	-0.9483 (1.9781)	1.5
	90 125	3.1623	0.10	90.07 (0.79) 125.06 (1.06)			2.1142 (0.7648)	-0.0241 (0.7118)	-0.0148 (0.7432)	-0.1772 (1.0389)	17.7	90.00 (0.00) 123.75 (1.42)			-0.1524 (1.4188)	-0.7904 (1.3054)	-0.0041 (2.4865)	0.4977 (2.0650)	0.8
	155 205	2.2361	0.05	154.30 (2.58) 204.88 (2.56)			1.8699 (0.8247)	0.0779 (0.8887)	0.2210 (0.9442)	-0.1537 (1.3397)	4.3	155.36 (6.92) 204.64 (3.65)			-0.2585 (2.0677)	0.5069 (1.6802)	0.7955 (1.7805)	-0.6782 (1.9195)	1.4
	180 235	3.8730	0.15	179.97 (0.79) 234.91 (0.92)			2.3014 (0.5465)	0.0103 (0.7031)	0.0005 (0.7007)	0.0740 (0.9851)	38.7	181.50 (3.37) 233.00 (6.75)			0.2875 (1.0961)	0.1172 (1.4326)	-0.6596 (1.2654)	-0.6498 (4.0957)	1.0
	40 275	3.1623	0.10	40.16 (1.25) 274.89 (1.45)			2.1290 (0.7526)	0.0801 (0.6789)	-0.0082 (0.7730)	-0.0998 (1.0386)	19.0	43.75 (4.79) 279.73 (13.00)			0.4742 (1.4951)	-0.1303 (0.4311)	-1.1829 (2.1184)	1.4819 (2.6986)	0.4
	75 220	3.8730	0.15	74.94 (1.25) 220.03 (0.80)			2.2883 (0.5359)	0.0244 (0.6756)	-0.0463 (0.6790)	-0.0572 (1.0426)	40.4	74.22 (7.44) 221.89 (5.33)			1.2061 (1.2377)	-0.2355 (0.5880)	-0.3273 (1.5663)	1.2515 (2.6408)	0.9
ad	False											20.00	1.6804 (0.6272)	-0.0157 (0.6827)					49.7
												40.00	2.1831 (0.7871)	-0.0611 (0.8366)					82.0
												75.00	2.6414 (1.1288)	-0.0105 (1.2091)					93.1
												90.00	2.2016 (1.3590)	0.0052 (1.4555)					82.9
												155.00	1.8083 (2.8044)	0.0904 (2.9215)					50.8
												180.00	2.6864 (2.4260)	0.0069 (2.5220)					92.6
	20 60	3.1623	0.05	20.06 (2.03) 60.01 (0.31)			-0.0132 (0.4323)	3.2295 (0.6212)	-0.0198 (0.6077)	-0.0048 (0.8326)	85.2	20.04 (1.54) 59.97 (1.86)			-0.0074 (0.5226)	2.9301 (1.4018)	0.0366 (0.6795)	-0.0288 (0.9225)	53.5
	90 125	4.4721	0.10	90.00 125.00			0.0093	4.3685	-0.0210	0.0296	99.6	89.92 125.10			0.0244	3.9697	-0.0185	-0.0431	85.4

					(0.18)	(0.20)	(0.4389)	(0.6991)	(0.5955)	(0.8432)		(2.81)	(2.50)			(0.5187)	(1.5257)	(0.7108)	(1.0203)	
	155	205	3.1623	0.05	155.12	204.99	-0.0305	3.2378	0.0314	0.0002	85.8	154.98	205.05			0.0009	2.9384	0.0787	-0.0418	55.8
					(2.24)	(0.25)	(0.4355)	(0.6142)	(0.5917)	(0.8570)		(2.07)	(4.24)			(0.5195)	(1.3717)	(0.6563)	(0.9010)	
	180	235	5.4772	0.15	180.00	235.00	-0.0103	5.3494	0.0194	0.0109	100.0	179.81	234.79			-0.0155	4.8139	0.0064	-0.1074	94.1
					(0.09)	(0.32)	(0.4613)	(0.7706)	(0.6096)	(0.8507)		(3.60)	(5.02)			(0.5646)	(1.7210)	(0.7435)	(1.0135)	
	40	275	4.4721	0.10	39.99	275.01	-0.0077	4.3919	-0.0247	-0.0322	99.2	39.97	274.99			-0.0047	4.0238	-0.0055	-0.1353	84.6
					(0.20)	(0.39)	(0.4450)	(0.7142)	(0.5963)	(0.8606)		(1.16)	(1.89)			(0.5312)	(1.5390)	(0.7020)	(0.9637)	
	75	220	5.4772	0.15	75.01	220.05	0.0086	5.4025	-0.0140	0.0409	100.0	74.87	220.00			0.0322	4.9274	-0.0151	-0.0806	93.7
					(0.42)	(0.97)	(0.4549)	(0.7895)	(0.5891)	(0.8292)		(3.27)	(1.97)			(0.5504)	(1.7417)	(0.7251)	(1.0327)	
												60.00		1.7408	0.0182					49.7
														(1.1939)	(1.3089)					
												125.00		2.2361	0.0195					81.8
														(1.8227)	(1.9220)					
			False									205.00		1.8385	0.1758					47.9
														(3.7903)	(3.9139)					
												225.00		2.6969	0.0132					91.9
														(2.9368)	(3.0488)					
												235.00		2.7493	0.0692					91.7
														(3.1465)	(3.2457)					
												275.00		2.3109	0.1243					81.4
														(3.8872)	(3.9899)					
da	20	60	3.1623	0.05	20.02	59.95	-0.0018	-0.0112	3.2509	-0.0295	85.6	20.19	59.93			0.0232	0.0081	2.9132	0.0033	53.0
					(0.42)	(2.06)	(0.4589)	(0.6015)	(0.6030)	(0.8144)		(3.62)	(3.41)			(0.5377)	(0.6681)	(1.4582)	(0.9348)	
	90	125	4.4721	0.10	90.00	125.01	-0.0015	-0.0067	4.4035	0.0209	99.6	90.01	124.96			-0.0019	-0.0106	4.0360	-0.0490	84.4
					(0.16)	(2.36)	(0.4596)	(0.6010)	(0.6629)	(0.8171)		(1.42)	(3.16)			(0.5319)	(0.7151)	(1.4779)	(0.9857)	
	155	205	3.1623	0.05	155.00	204.96	-0.0013	0.0069	3.1922	-0.0065	84.0	155.04	204.77			0.0054	-0.0106	2.9291	-0.0611	55.0
					(1.53)	(1.29)	(0.4342)	(0.6074)	(0.6132)	(0.8882)		(1.71)	(4.86)			(0.5137)	(0.7391)	(1.3285)	(1.0065)	
	180	235	5.4772	0.15	179.99	235.00	-0.0248	-0..0010	5.3695	0.0501	100.0	180.00	234.86			0.0273	0.0364	4.9181	-0.0710	93.8
					(0.13)	(0.13)	(0.4637)	(0.5881)	(0.7347)	(0.8678)		(0.57)	(2.47)			(0.5535)	(0.7547)	(1.6764)	(1.0174)	
	40	275	4.4721	0.10	39.98	274.99	-0.0115	0.0166	4.3995	0.0086	99.5	39.95	274.86			-0.0063	-0.0066	4.0022	-0.0710	84.8
					(0.85)	(0.25)	(0.4542)	(0.6058)	(0.6849)	(0.8223)		(1.64)	(5.98)			(0.5263)	(0.7317)	(1.5275)	(1.0116)	
	75	220	5.4772	0.15	75.00	220.00	0.0026	0.0102	5.3781	0.0470	100.0	74.99	219.98			-0.0086	-0.0018	4.9175	-0.0642	93.2
					(0.35)	(0.13)	(0.4674)	(0.6022)	(0.7459)	(0.8616)		(1.51)	(0.42)			(0.5637)	(0.7858)	(1.6850)	(1.0590)	
	20	60	4.4721	0.05	20.00	59.98	0.0195	0.0044	-0.0052	4.5075	84.3	19.99	59.88			0.0527	0.0261	-0.0319	4.3680	60.8
					(1.02)	(0.44)	(0.4281)	(0.5910)	(0.6068)	(0.8971)		(0.46)	(1.95)			(0.4498)	(0.6478)	(0.6291)	(1.0112)	
	90	125	6.3246	0.10	90.00	125.03	0.0118	0.0070	0.0102	6.1925	99.6	90.01	124.99			-0.0162	0.0039	-0.0159	5.7160	95.4
					(0.17)	(0.62)	(0.4580)	(0.6165)	(0.5923)	(1.0142)		(0.81)	(0.59)			(0.4767)	(0.6299)	(0.6617)	(1.1258)	
	155	205	4.4721	0.05	155.03	205.02	-0.0013	-0.0003	0.0123	4.5532	84.8	155.02	204.99			-0.0098	-0.0186	0.0130	4.4067	64.1
					(0.46)	(0.49)	(0.4525)	(0.6014)	(0.5713)	(0.8621)		(1.41)	(0.35)			(0.4481)	(0.6484)	(0.6094)	(1.0515)	
dd	180	235	7.7460	0.15	180.00	235.02	-0.0133	0.0021	0.0294	7.5505	100.0	180.02	235.02			-0.0058	0.0082	0.0290	6.9661	99.1
					(0.21)	(0.60)	(0.4835)	(0.6162)	(0.6108)	(1.1286)		(0.35)	(1.21)			(0.4937)	(0.6737)	(0.6646)	(1.2964)	
	40	275	6.3246	0.10	40.02	274.98	0.0032	0.0383	-0.0103	6.2165	99.5	40.03	274.98			-0.0066	0.0243	0.0012	5.7631	95.3
					(0.58)	(0.40)	(0.4717)	(0.6241)	(0.6094)	(1.0482)		(0.62)	(0.54)			(0.4843)	(0.6720)	(0.6500)	(1.1763)	
	75	220	7.7460	0.15	74.99	219.99	-0.0032	-0.0191	0.0155	7.5711	100.0	75.00	220.02			0.0145	-0.0342	0.0014	6.9677	99.2
					(0.21)	(0.14)	(0.4972)	(0.5998)	(0.6146)	(1.1307)		(0.59)	(0.57)			(0.4856)	(0.6447)	(0.6642)	(1.3232)	

* The numbers in parentheses are the standard deviations of 1000 replications. The sample size was equal to 240. The same is true for the later Tables.

Table S3 | Comparisons of trait phenotype, general combining ability (GCA), specific combining ability (SCA) and mid-parental heterosis (MPH) in the genetic dissection of heterosis under partial-, complete-, and over-dominance models using Monte Carlo simulation experiments

QTL1		*d*=0.5*a*				*d*=*a*				*d*=2*a*			
		Posi (cM)	*a*	*d*	r^2	Posi (cM)	*a*	*d*	r^2	Posi (cM)	*a*	*d*	r^2
True value		25	1.4907	0.7454	0.05	25	1.2910	1.2910	0.05	25	0.9129	1.8257	0.05
Trait phenotype	Estimate	24.99	1.5632	0.6934		25.02	1.0888	1.0545		25.09	0.7256	1.7099	
	SD	0.18	0.4009	0.4647		0.49	1.0466	0.8710		0.99	0.9099	0.8784	
	Power(%)	66.3				43.2				50.5			
GCA	Estimate	25.00	0.7298	-0.0024		24.99	0.6350	-0.0029		25.00	0.4496	-0.0051	
	SD	0.00	0.0543	0.0323		0.09	0.0662	0.0271		0.00	0.0633	0.0200	
	Power (%)	100.0				100.0				100.0			
SCA	Estimate	24.52	0.0051	1.6513		25.27	0.0676	1.7220		24.99	0.0232	1.9997	
	SD	2.18	0.3768	0.3858		1.55	0.3031	0.6905		0.11	0.3094	0.4000	
	Power (%)	2.1				9.2				54.9			
Mid-parent heterosis	Estimate	25.38	-0.0511	1.4640		25.18	-0.1152	2.2374		24.94	-0.0547	2.4434	
	SD	3.20	1.1177	1.1086		1.44	0.4359	0.5727		1.04	0.4413	0.7005	
	Power (%)	1.3				4.9				21.2			
QTL2		Posi (cM)	*a*	*d*	r^2	Posi (cM)	*a*	*d*	r^2	Posi (cM)	*a*	*d*	r^2
True value		75	2.1082	1.0541	0.10	75	1.8257	1.8257	0.10	75	1.2910	2.5820	0.10
Trait phenotype	Estimate	75.00	2.0283	0.9304		74.96	1.3947	1.2668		75.01	0.7923	1.9952	
	SD	0.14	0.4816	0.4264		0.53	0.7438	0.6092		0.53	0.6763	0.9143	
	Power (%)	95.7				79.3				81.3			

GCA	Estimate	75.00	1.0329	-0.0024		75.00	0.8958	-0.0029		75.00	0.6359	-0.0051	
	SD	0.00	0.0774	0.0323		0.01	0.0844	0.0271		0.00	0.0884	0.0200	
	Power (%)	100.0				100.0				100.0			
SCA	Estimate	74.67	-0.0769	1.7465		75.04	-0.0010	2.0765		75.00	-0.0014	2.5790	
	SD	2.18	0.3788	0.3864		0.56	0.3084	0.4083		0.12	0.3184	0.4594	
	Power (%)	7.5				45.5				92.2			
Mid-parent heterosis	Estimate	74.47	-0.0743	1.9853		75.00	-0.0477	2.6145		75.00	-0.0779	3.0142	
	SD	2.26	0.5779	0.7917		0.00	0.4120	0.4394		0.10	0.4195	0.5689	
	Power (%)	3.8				22.1				63.4			
QTL3		Posi (cM)	*a*	*d*	r^2	Posi (cM)	*a*	*d*	r^2	Posi (cM)	*a*	*d*	r^2
True value		135	1.4907	0.7454	0.05	135	1.2910	1.2910	0.05	135	0.9129	1.8257	0.05
Trait phenotype	Estimate	134.99	1.5813	0.6895		134.86	1.2246	1.0287		135.01	1.2290	1.8104	
	SD	0.12	0.4825	0.4773		1.19	1.0158	0.7531		0.56	0.8942	0.8360	
	Power (%)	64.9				44.8				45.1			
GCA	Estimate	135.00	0.7280	-0.0024		135.00	0.6306	-0.0029		135.00	0.4464	-0.0051	
	SD	0.00	0.0593	0.0323		0.00	0.0613	0.0271		0.00	0.0660	0.0200	
	Power (%)	100.0				100.0				100.0			
SCA	Estimate	135.00	0.4503	1.4810		135.08	0.0708	1.7970		135.01	0.0115	2.0433	
	SD	1.25	0.2604	0.4830		0.91	0.2916	0.5485		0.14	0.2931	0.3655	
	Power (%)	1.6				12.1				53.8			
Mid-parent heterosis	Estimate	136.82	-0.0433	1.7546		134.78	0.0125	2.4241		135.04	-0.0497	2.5849	
	SD	4.05	0.8944	1.0154		1.49	0.4098	0.4072		0.65	0.4336	0.5117	

	Power (%)	0.9				4.5				23.7			
QTL4		Posi (cM)	*a*	*d*	r^2	Posi (cM)	*a*	*d*	r^2	Posi (cM)	*a*	*d*	r^2
True value		175	2.5820	1.2910	0.15	175	2.2361	2.2361	0.15	175	1.5811	3.1623	0.15
Trait phenotype	Estimate	175.01	2.4041	1.0861		174.97	1.5055	1.4016		174.99	0.9701	2.1122	
	SD	0.17	05864	0.4060		1.40	0.7792	0.5759		0.50	0.5598	0.9275	
	Power (%)	99.4				92.2				95.7			
GCA	Estimate	175.00	1.2647	-0.0024		175.00	1.0934	-0.0029		175.00	0.7772	-0.0051	
	SD	0.00	0.0964	0.0323		0.02	0.1078	0.0271		0.00	0.1138	0.0200	
	Power (%)	100.0				100.0				100.0			
SCA	Estimate	175.00	-0.0170	1.8211		175.01	0.0219	2.3449		175.00	-0.0012	3.0905	
	SD	0.00	0.2900	0.2623		0.35	0.3328	0.4185		0.06	0.3449	0.5100	
	Power (%)	19.1				75.0				99.6			
Mid-parent heterosis	Estimate	174.87	-0.0386	2.1653		174.96	-0.0923	2.7376		175.00	-0.1102	3.4635	
	SD	0.80	0.4319	0.6966		0.74	0.4425	0.5311		0.17	0.4366	0.6505	
	Power (%)	7.7				50.0				88.3			
QTL5		Posi (cM)	*a*	*d*	r^2	Posi (cM)	*a*	*d*	r^2	Posi (cM)	*a*	*d*	r^2
True value		220	2.1082	1.0541	0.10	220	1.8257	1.8257	0.10	220	1.2910	2.5820	0.10
Trait phenotype	Estimate	219.87	2.0419	0.9348		219.70	1.3928	1.2705		220.00	0.8752	1.9730	
	SD	4.08	0.4727	0.4276		8.42	0.7582	0.5952		0.54	0.6397	0.9100	
	Power (%)	94.6				77.5				81.2			
GCA	Estimate	220.00	1.0317	-0.0024		220.00	0.8957	-0.0029		220.00	0.6337	-0.0051	
	SD	0.00	0.0801	0.0323		0.01	0.0787	0.0271		0.00	0.0902	0.0200	

	Power (%)	100.0					100.0					100.0			
SCA	Estimate	219.94	0.0414	1.7468			219.98	0.0143	2.1208			220.00	0.0122	2.5688	
	SD	1.21	0.3352	0.3899			0.28	0.3186	0.4026			0.00	0.3320	0.4536	
	Power (%)	8.6					43.4					91.8			
Mid-parent heterosis	Estimate	220.19	-0.0678	2.1797			220.03	-0.0569	2.5631			219.99	-0.0748	3.0314	
	SD	0.96	0.4954	0.3541			0.34	0.4653	0.5135			0.10	0.4218	0.5809	
	Power (%)	2.7					23.9					61.0			
QTL6		Posi (cM)	*a*	*d*	r^2		Posi (cM)	*a*	*d*	r^2		Posi (cM)	*a*	*d*	r^2
True value		270	2.5820	1.2910	0.15		270	2.2361	2.2361	0.15		270	1.5811	3.1623	0.15
Trait phenotype	Estimate	270.00	2.3977	1.0645			269.92	1.4552	1.3944			269.98	0.9576	2.0999	
	SD	0.07	0.5897	0.4134			2.60	0.7785	0.5948			0.43	0.5729	0.9317	
	Power (%)	99.4					92.9					94.4			
GCA	Estimate	270.00	1.2662	-0.0024			270.00	1.0934	-0.0029			270.00	0.7725	-0.0051	
	SD	0.00	0.0975	0.0323			0.03	0.1053	0.0271			0.00	0.1106	0.0200	
	Power (%)	100.0					100.0					100.0			
SCA	Estimate	270.03	0.0325	1.8047			270.02	-0.0141	2.3334			270.00	0.0116	3.0958	
	SD	0.65	0.2968	0.4344			0.37	0.3271	0.4449			0.00	0.3432	0.5159	
	Power (%)	17.7					76.6					98.2			
Mid-parent heterosis	Estimate	269.87	-0.0325	2.2862			270.00	-0.0935	2.7613			270.00	-0.1000	3.4680	
	SD	1.13	0.4408	0.3967			0.67	0.4291	0.5139			0.00	0.4362	0.6241	
	Power (%)	7.8					47.7					88.5			

Table S4 | Genetic basis of heterosis from 2,160,000 simulated F_1 individuals

QTL	Heterosis			r^2	Power of QTL detection from phenotypic observation					
	Absolute of Mean (SD)	95% Confidence interval	CV		*a*	*d*	*a*×*a*	*a*×*d*	*d*×*a*	*d*×*d*
Additive (*a*)	0.0772 (0.2785)	-7.5097~7.9917	72.47	0.05	85.0					
				0.10	99.5					
				0.05	85.7					
				0.15	99.8					
				0.10	99.4					
				0.15	100.0					
Dominance (*d*)	10.28621 (0.4402)	-1.7238~22.6488	0.60	0.05		87.2				
				0.10		99.9				
				0.05		86.6				
				0.15		100.0				
				0.10		99.9				
				0.15		100.0				
a×*a*	0.1851 (0.4317)	-10.1961~11.8511	-2.32	0.05			85.4			
				0.10			99.4			
				0.05			85.2			
				0.15			100.0			
				0.10			99.1			
				0.15			99.9			
a×*d*	0.2008 (0.5303)	-12.7127~14.3138	-8.03	0.05				86.1		
				0.10				99.8		
				0.05				88.5		
				0.15				100.0		
				0.10				99.9		
				0.15				100.0		
d×*a*	0.1689 (0.5141)	-12.7198~14.2701	-3.78	0.05					85.8	
				0.10					99.7	
				0.05					85.4	
				0.15					100.0	
				0.10					99.8	
				0.15					100.0	
d×*d*	8.4467 (0.3425)	-18.1604~1.4647	-0.59	0.05						86.0
				0.10						99.8
				0.05						88.2
				0.15						100.0
				0.10						99.6
				0.15						100.0
d=0.5*a*	3.2542 (0.3184)	-5.0630~11.9028	1.3203	0.05	66.3	66.3				
				0.10	95.7	95.7				
				0.05	64.9	64.9				
				0.15	99.4	99.4				
				0.10	94.6	94.6				
				0.15	99.4	99.4				
d=2*a*	8.2501 (0.3945)	-2.4574~19.3589	0.6670	0.05	50.5	50.5				
				0.10	81.3	81.3				
				0.05	45.1	45.1				
				0.15	95.7	95.7				
				0.10	81.2	81.2				
				0.15	94.4	94.4				
d=*a*	5.7222 (0.3474)	-3.6537~15.4374	0.8410	0.05	43.2	43.2				
				0.10	79.3	79.3				
				0.05	44.8	44.8				
				0.15	92.2	92.2				
				0.10	77.5	77.5				
				0.15	92.9	92.9				

Table S5 | Comparison of trait phenotype and mid-parental heterosis (MPH) under additive, dominance, *aa*, *ad*, *da*, and *dd* effects in F_2

QTL	Type	True value				Estimates from trait phenotype				Estimates from MPH			
		Posi. (cM)		Effect	r^2	Posi. (cM)		Effect	Power (%)	Posi. (cM)		Effect	Power (%)
1	Additive (*a*)	70		0.7071	0.10	70.10 (2.11)		0.7048 (0.0969)	83.9	70.09 (2.09)		0.6976 (0.0962)	83.9
2	Dominance (*d*)	145		1.000	0.10	144.96 (1.02)		0.9911 (0.1367)	100.0	144.97 (0.99)		0.9818 (0.1357)	100.0
3	$a \times a$	35	285	1.000	0.10	34.93 (2.57)	284.98 (2.33)	0.9818 (0.1418)	97.0	34.93 (2.57)	284.98 (2.34)	0.9730 (0.1392)	96.9
4	$a \times d$	95	170	1.4142	0.10	94.60 (1.69)	169.98 (1.23)	1.3981 (0.1977)	99.5	94.60 (1.69)	169.98 (1.20)	1.3834 (0.1966)	99.5
5	$d \times a$	120	235	1.4142	0.10	120.00 (1.74)	235.00 (2.30)	1.3935 (0.1893)	99.0	120.00 (1.74)	235.00 (2.30)	1.3800 (0.1874)	98.9
6	$d \times d$	195	255	2.000	0.10	194.93 (0.63)	254.98 (1.08)	1.9738 (0.2676)	99.7	194.93 (0.63)	254.98 (1.07)	1.9538 (0.1712)	99.7

*The sample size was equal to 300.

Table S6 | The true and estimated values for QTL parameters using real molecular marker dataset and the 0.01 significance level

QTL	QTL Type	True value			Estimate															
					Trait phenotype								GCA							
		Marker	Posi (cM)	Effect	Posi (cM)	*a*	*d*	*aa*	*ad*	*da*	*dd*	Power (%)	Posi (cM)	*a*	*d*	*aa*	*ad*	*da*	*dd*	Power (%)
QTL1	*ad*	A4-1 D2-1	18.34 23.60	0.40	18.34 (0.00) 23.60 (0.00)			-0.0199<br>(0.1193)	0.3395<br>(0.2209)	-0.0241<br>(0.2021)	-0.0573<br>(0.3857)	100.0	18.34 (0.00) 23.60 (0.00)			-0.0449<br>(0.0129)	-0.0102<br>(0.0130)	-0.0018<br>(0.0118)	0.0172<br>(0.0052)	8.5
QTL2	*ad*	A5-1 A5-2	26.14 95.80	0.44	26.14 (0.00) 95.80 (0.00)			-0.0190<br>(0.1205)	0.3736<br>(0.2299)	-0.0245<br>(0.2093)	-0.0605<br>(0.4003)	99.9	26.14 (0.00) 95.80 90.00)			-0.0460<br>(0.0147)	-0.0097<br>(0.0103)	-0.0008<br>(0.0094)	0.0167<br>(0.0053)	22.5

QTL	QTL Type	True value			SCA								MPH							
		Marker	Posi (cM)	Effect	Posi (cM)	*a*	*d*	*aa*	*ad*	*da*	*dd*	Power (%)	Posi (cM)	*a*	*d*	*aa*	*ad*	*da*	*dd*	Power (%)
QTL1	False QTL												18.34	4.1655<br>(0.9544)	-2.4131<br>(1.4016)					6.9
QTL2	False QTL												26.14	4.4357<br>(0.8507)	-2.6822<br>(1.5639)					8.2
QTL1	*ad*	A4-1 D2-1	18.34 23.60	0.40	18.34 (0.00) 23.60 (0.00)			0.2670<br>(0.1612)	0.1095<br>(0.0758)	0.0912<br>(0.0746)	-0.5172<br>(0.2113)	66.5	18.34 (0.00) 23.60 (0.00)			-0.5443<br>(2.4514)	5.6387<br>(2.2560)	-0.6636<br>(2.3172)	-3.5870<br>(3.4101)	57.8
QTL2	*ad*	A5-1 A5-2	26.14 95.80	0.44	26.14 (0.00) 95.80 (0.00)			0.2897<br>(0.1681)	0.1175<br>(0.0749)	0.0965<br>(0.0736)	-0.5522<br>(0.2197)	74.0	26.14 (0.00) 95.80 (0.00)			-0.6112<br>(2.3727)	5.9524<br>(2.3402)	-0.5894<br>(2.4100)	-3.9025<br>(3.4834)	60.6

The software GAS_NCII (Genetic Analysis System for NC II mating design):

The Windows software in this study was attached (To first install JAVA Runtime Environment (jdk-7u71-windows-x64.exe) by default install directory, and to then install Maltlab Runtime Environment (R2014b (8.4) For Windows). The two files are too large. Readers may download the first file at http://www.oracle.com/technetwork/java/javase/downloads/jdk7-downloads-1880260.html and the second file at http://cn.mathworks.com/products/compiler/mcr/index.html, respectively). If the readers want to use another jdk version please change your jdk directory in run.bat file. The above two files are also downloaded from http://yun.baidu.com/share/link?shareid=2858238674&uk=3276116826. EMS memory size in the running computer is more than 8 G. By running "run.dat" file, prior parameters *a* and *b* need to be determined by cross-validation experiments. Thus, our suggestion is to run the software several times and select the best results.

Maternal	Paternal									
	F_1	F_2	F_3	F_4	F_5	F_6	…	…	F_{79}	F_{80}
M_1	*									
M_2		*								
M_3			*							
M_4				*						
M_5					*					
…						*				
…							…			
…								*		
M_{79}									*	
M_{80}										*

I

Maternal	Paternal									
	F_1	F_2	F_3	F_4	F_5	F_6	…	…	F_{47}	F_{48}
M_1	*	*	*							
M_2		*	*	*						
M_3			*	*	*					
M_4				*	*	*				
M_5					*	*	*			
…						*	…			
…							…			
…								*	*	*
M_{47}								*	*	*
M_{48}								*	*	*

II

Maternal	Paternal									
	F_1	F_2	F_3	F_4	F_5	F_6	…	…	F_{33}	F_{34}
M_1	*	*	*	*	*					
M_2		*	*	*	*	*				
M_3			*	*	*	*	*			
M_4				*	*	*	*	*		
M_5					*	*	*	*	*	
…						*	…			
…							…			
…							*	*	*	*
M_{33}									…	
M_{34}							*	*	*	*

III

Maternal	Paternal									
	F_1	F_2	F_3	F_4	F_5	F_6	F_7	F_8	…	F_{15}
M_1	*	*	*	*	*	*	*	*	*	*
M_2	*	*	*	*	*	*	*	*	*	*
M_3	*	*	*	*	*	*	*	*	*	*
M_4	*	*	*	*	*	*	*	*	*	*
M_5	*	*	*	*	*	*	*	*	*	*
M_6	*	*	*	*	*	*	*	*	*	*
M_7	*	*	*	*	*	*	*	*	*	*
M_8	*	*	*	*	*	*	*	*	*	*
…	*	*	*	*	*	*	*	*	*	*
M_{15}	*	*	*	*	*	*	*	*	*	*

IV

Figure S1 | Mating strategies in NCII genetic mating design. In the NCII mating design, each maternal line was crossed with one (I), two, three (II), four, five (III), six, seven, and fifteen (IV) paternal lines.

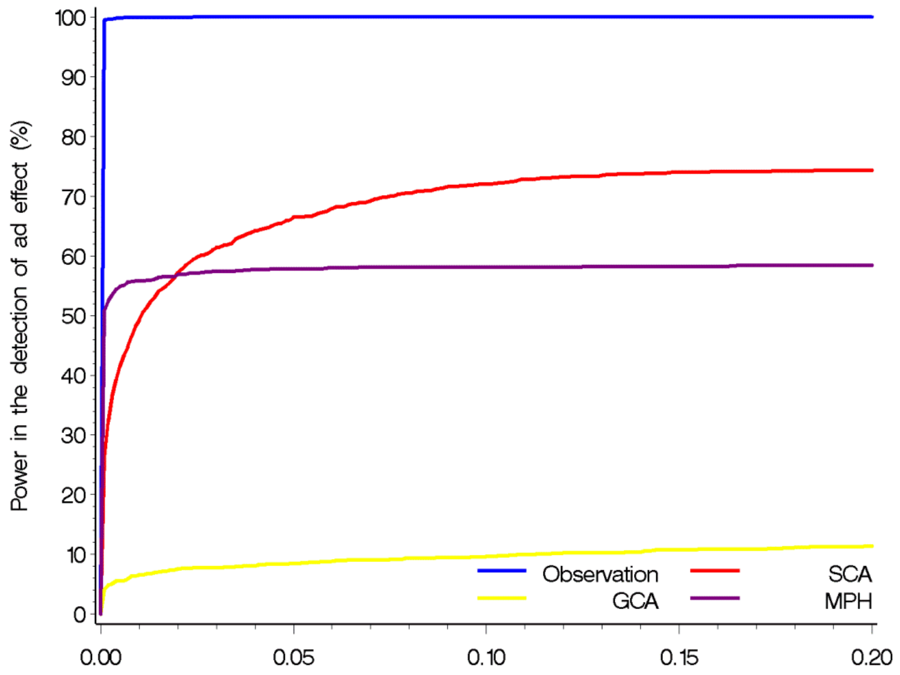

A

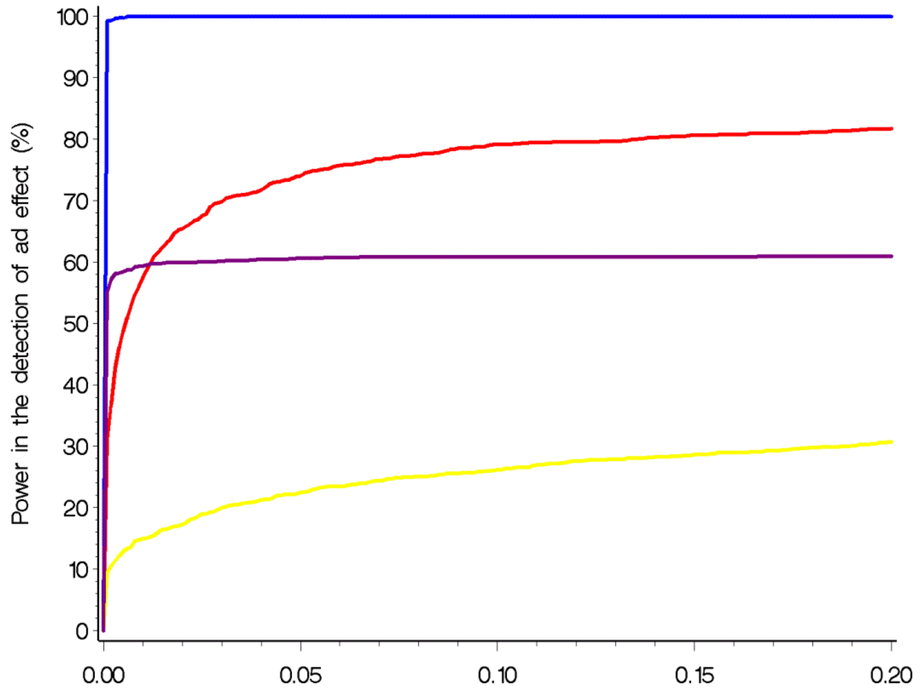

B

Figure S2 | Statistical power in the detection of QTL1 (A) and QTL2 (B) under different false discovery rates for various phenotypic measures (trait phenotype(observation), GCA, SCA and MPH). The true and estimated values for simulated QTL are listed in Table S6.
